# Periodontitis and Endotoxemia: Clinical and Microbiological Insights

**DOI:** 10.1111/jcpe.70148

**Published:** 2026-06-04

**Authors:** Anbo Dong, Muhammed Manzoor, Jaakko Leskelä, Jukka Putaala, Eija Könönen, Yi Jin, Pirkko Pussinen, Susanna Paju, Svetislav Zaric

**Affiliations:** ^1^ Centre for Host‐Microbiome Interactions, Faculty of Dentistry, Oral & Craniofacial Sciences King's College London London UK; ^2^ Department of Oral and Maxillofacial Diseases University of Helsinki Helsinki Finland; ^3^ School of Medicine, Institute of Dentistry University of Eastern Finland Kuopio Finland; ^4^ Department of Neurology Helsinki University Hospital and University of Helsinki Helsinki Finland; ^5^ Institute of Dentistry University of Turku Turku Finland

**Keywords:** dysbiosis, endotoxemia, lipopolysaccharide, periodontitis, subgingival plaque

## Abstract

**Aim:**

Endotoxemia has been proposed as a link between periodontitis and systemic complications through the translocation of lipopolysaccharide (LPS) from inflamed periodontal sites into the bloodstream. This study aimed to investigate subgingival and serum LPS activities and their correlations with periodontal clinical and microbiological parameters.

**Materials and Methods:**

Two‐hundred and seventy participants with gingivitis and periodontitis from the SECRETO Oral cohort were included in the study. Serum and subgingival plaque samples were analysed for LPS activity using the recombinant Factor C assay, and subgingival microbiota were profiled by 16S rRNA sequencing. Dysbiosis indices and taxonomic associations were assessed through multivariate regressions and MaAsLin2 models adjusted for clinical and demographic covariates. Functional pathways were inferred using PICRUSt2.

**Results:**

Serum LPS activities were significantly elevated in periodontitis patients compared to gingivitis patients and correlated with the subgingival microbial dysbiosis index (SMDI). Subgingival LPS was also significantly higher in periodontitis patients and showed strong associations with subgingival bacterial load and SMDI. Subgingival LPS explained 10% of serum LPS variations. Differential abundance analysis identified *Fretibacterium* as positively associated with both subgingival and serum LPS activities. Functional prediction revealed enrichment of bacterial motility and invasion pathways, with depletion of host defence functions, supporting a mechanistic link between subgingival dysbiosis, LPS translocation and systemic endotoxemia.

**Conclusion:**

These findings provide novel evidence that elevated serum LPS activities are closely linked with periodontitis diagnosis, associated with subgingival dysbiosis, and highlight the periodontal niche as a source of systemic endotoxemia. In addition, it identified *Fretibacterium* as the main contributor to both subgingival and serum LPS activities.

## 1 Introduction

Periodontitis is a chronic, dental biofilm–induced inflammatory condition that not only leads to progressive destruction of tooth‐supporting structures but is also increasingly recognised as a systemic health modifier (Papapanou et al. 2018). It has been associated with a range of extra‐oral diseases, including cardiovascular disease, diabetes, rheumatoid arthritis and adverse pregnancy outcomes (Kapila 2021). These associations are thought to be mediated by translocated microbial components and sustained inflammatory responses, implicating periodontitis as a potential contributor to systemic disease processes through both immunological and microbial pathways (Pietiäinen et al. 2018; Pussinen et al. 2022).

Endotoxemia, defined as the presence of lipopolysaccharide (LPS) in the bloodstream, is a key mediator of systemic low‐grade inflammation. In most cases, the gastrointestinal tract has been considered the principal source of circulating LPS (Moludi et al. 2020). High‐fat diets, gut dysbiosis and epithelial barrier defects facilitate microbial translocation, contributing to cardiometabolic diseases and systemic inflammation (Pussinen et al. 2022).

However, the gut‐centric view does not fully explain alternative sources of serum endotoxin. In periodontitis, the subgingival niche is highly vascularised and chronically inflamed, with epithelial integrity frequently compromised (Meyle and Chapple 2015). The ulcerated epithelium within periodontal pockets, compounded by mechanical stimulation during routine activities such as mastication or toothbrushing, may facilitate translocation of bacterial endotoxins from subgingival areas into the systemic circulation (Vitkov et al. 2023). Moreover, pathobionts, such as *Porphyromonas gingivalis* , produce atypical LPS with under‐acylation and altered phosphorylation patterns (Jiménez et al. 2022; Zhang et al. 2021), which attenuate Toll‐like receptor recognition, evade immune surveillance and enhance paracellular passage (Coats et al. 2009; Kroemer et al. 2024). These features render the inflamed periodontal pockets not only a localised problem but also plausible contributors to systemic endotoxin burden.

Previous studies have examined the link between periodontal inflammation and systemic endotoxin burden, providing early evidence for an oral source of endotoxemia. Salivary LPS activity has been associated with cardiovascular risk and serum LPS levels, and immune responses to periodontal pathogens have predicted systemic inflammatory and vascular outcomes (Liljestrand et al. 2017; Pussinen et al. 2007). It has also been linked to specific microbial and functional signatures within the oral microbiome (Manzoor et al. 2025). Meanwhile, Shaddox et al. (2011) reported that serum LPS levels correlated with periodontal severity in localised aggressive periodontitis, supporting an oral contribution to systemic endotoxemia.

Although previous studies have examined LPS levels in saliva and serum, the relationship between clinical periodontal parameters, subgingival microbiota and LPS activity and systemic endotoxin burden remains unclear. Specific microbial taxa that drive subgingival LPS activity and their potential contributions to circulating LPS levels have not been systematically characterised. Furthermore, it is not known whether such microbial–LPS associations differ between gingivitis and periodontitis patients. These knowledge gaps hinder a more integrated understanding of how periodontal dysbiosis may contribute to systemic inflammation.

The aim of this study was to investigate serum LPS activities in patients with periodontitis and gingivitis, and to identify clinical parameters and subgingival microbiome characteristics that contribute to local and systemic endotoxin burden.

## 2 Materials and Methods

### 2.1 Study Design and Participants

This was a retrospective analysis of 270 SECRETO Oral cohort (NCT01934725) participants, age 18–49, recruited at Helsinki and Turku University Hospitals (Finland) between 2013 and 2019. Ethical approval was obtained from the Ethics Committees of Helsinki University Hospital (ETH11808) and Turku University Central Hospital (STE04294), and all participants provided written informed consent.

The primary outcome of this study was serum endotoxin activity, while secondary outcomes included subgingival endotoxin activity and subgingival microbial dysbiosis index (SMDI).

Full‐mouth clinical and radiographic examinations were performed by a single calibrated periodontal specialist. Periodontal diagnoses were assigned according to the 2017 World Workshop Classification and categorised as clinical periodontal health (PPD ≤ 3 mm and BOP < 10%, only 1 participant), gingivitis (BOP > 10% without clinical attachment or bone loss, 200 participants) or periodontitis (70 participants). Periodontitis was defined by the presence of interdental clinical attachment loss (CAL) at ≥ 2 non‐adjacent teeth, or buccal/oral CAL ≥ 3 mm with probing pocket depth (PPD) ≥ 3 mm at ≥ 2 teeth, in the absence of non‐periodontal causes (Papapanou et al. 2018; Tonetti et al. 2018). Radiographic evidence of alveolar bone loss was used to support clinical diagnoses, staging and grading of periodontitis.

Participants were excluded if they were pregnant, had used systemic antibiotics within the previous 3 months or had systemic conditions known to affect periodontal health, except diabetes, which was retained as a covariate.

A recruitment flowchart is provided in Figure S1. Of the 324 recruited individuals, 290 yielded valid 16S rRNA sequencing data. After excluding 19 participants due to recent antibiotic use, 271 participants were retained in the dataset. Because only one participant met the criteria for periodontal health, this individual was not included in comparative analyses, and all subsequent analyses were restricted to comparisons between gingivitis and periodontitis groups (*n* = 270).

### 2.2 Sample Collection and Laboratory Procedures

Subgingival plaque samples were collected from one representative sulcus or pocket per quadrant using a sterile Gracey periodontal curette, following periodontal probing during the full‐mouth examination. In gingivitis patients, samples were obtained from bleeding sites, whereas in periodontitis patients they were obtained from the deepest pockets exhibiting BOP. At each site, subgingival plaque was collected by inserting the curette into the sulcus or pocket and performing a single stroke along the root surface to ensure consistency of sampling. Samples from all sites were pooled for each participant, eluted in 500 μL of endotoxin‐free sterile water and stored at −80°C until processing.

DNA was extracted from a 300‐μL aliquot and quantified by qPCR using the Femto Bacterial DNA Quantification Kit (Zymo Research). The extracted DNA was also used for 16S rRNA gene sequencing to characterise microbial composition. The remaining amount was used for endotoxin activity measurement.

Peripheral blood samples were collected before periodontal examination and stored at −80°C until analysis. Endotoxin activity in serum and pooled subgingival plaque samples was quantified using a recombinant Factor C–based assay (ENDOZYME, bioMérieux, France) according to the manufacturer's instructions. All assays were performed in duplicates. Endotoxin activity was measured as a functional readout of biologically active endotoxin rather than total LPS mass concentration (Abate et al. 2017; Piehler et al. 2020).

### 2.3 Microbiome Sequencing and Data Analysis

Subgingival plaque samples were characterised by 16S rRNA gene sequencing targeting the V1–V2 region, enabling consistent profiling of key subgingival taxa. DNA extraction, sequencing and downstream bioinformatic processing were performed as previously described (Manzoor et al. 2025). Functional profiles were inferred using PICRUSt2 (Douglas et al. 2020). SMDI (Chen et al. 2022) was calculated as the mean CLR‐transformed difference between predefined dysbiotic and normobiotic genera (Table S1). Further bioinformatic and functional inference details are provided in Supplementary Methods.

### 2.4 Statistical Analysis

All statistical analyses were performed in R (v4.3.1). All analyses were conducted by comparing the gingivitis and periodontitis groups. Continuous variables were compared using Welch's *t*‐test or the Wilcoxon rank‐sum test, as appropriate according to data distribution. Categorical variables were analysed using the *χ* ^2^ or Fisher's exact test, as appropriate. Subgroup comparisons across periodontitis stages or grades were performed using the Kruskal–Wallis test. Multivariate linear regression models were used to assess associations of serum and subgingival LPS activity with clinical and demographic factors, adjusting for age, sex, smoking status, body mass index, diabetes and periodontal pocket measures (≥ 4 mm and ≥ 6 mm sites). Associations between LPS activity and microbial parameters were evaluated using Spearman correlations within diagnostic groups, with partial correlations adjusted for age, sex, smoking status and body mass index where indicated. Alpha diversity (observed richness, Shannon, Simpson) was compared between groups using Welch's *t*‐test or the Wilcoxon rank‐sum test, as appropriate, and beta diversity was assessed using PERMANOVA and visualised by principal coordinate analysis (PCoA). Differential abundance analysis between gingivitis and periodontitis groups was performed at the genus level using ANCOM‐BC2, with false discovery rate (FDR) adjustment applied for multiple testing. Genus‐level associations with LPS activity were assessed using multivariable linear models implemented in MaAsLin2, adjusted for age, sex, smoking status, diabetes and body mass index.

## 3 Results

### 3.1 Demographic and Clinical Characteristics of the Study Population

A total of 270 participants were included in the analyses, comprising 41 participants with localised gingivitis, 159 with generalised gingivitis and 70 with periodontitis. Group‐wise comparisons of demographic, clinical and microbial characteristics between gingivitis and periodontitis patients are summarised in Table 1.

**TABLE 1:** Demographic and clinical characteristics of participants.

	Gingivitis	Periodontitis	*p*
Number of participants	200	70	
Age (years), mean ± SD	38.7 ± 8.0	42.9 ± 6.0	**< 0.001**
Sex, female/male (%)	87/113 (43.5%/56.5%)	17/53 (24.3%/75.7%)	**0.007**
Current smoking, yes/no (%)	40/160 (20%/80%)	26/43 (37.7%/62.3%)	**0.004**
BMI (kg/m^2^), mean ± SD	26.7 ± 5.1	27.8 ± 5.0	0.071
Diabetes, yes/no (%)	3/197 (1.5%/98.5%)	3/67 (4.3%/95.7%)	0.182
Clinical			
Full‐mouth bleeding score (FMBS) (%), mean ± SD	38.7 ± 13.7	42.5 ± 15.3	0.060
Number of periodontal pockets (4–5 mm)	5.1 ± 5.8	10.5 ± 11.9	**0.003**
Number of periodontal pockets (≥ 6 mm)	0.3 ± 0.6	2.0 ± 7.6	**0.001**

*Note:* Bold values indicate statistically significant.

Although all participants were relatively young, individuals with periodontitis were significantly older than those without periodontitis (mean age: 42.9 ± 6.0 vs. 38.7 ± 8.0 years, *p* < 0.001). The periodontitis group also included a higher proportion of males and current smokers (both *p* < 0.01). Diabetes prevalence and BMI were not significantly different between the groups.

Regarding periodontal clinical parameters, the number of periodontal pockets measuring 4–5 mm and ≥ 6 mm was also higher in the periodontitis group (*p* = 0.003 and *p* = 0.001, respectively). Full‐mouth bleeding score (FMBS) was not statistically different between the groups (*p* = 0.060).

### 3.2 Comparison of Serum LPS Activity

**FIGURE 1:**
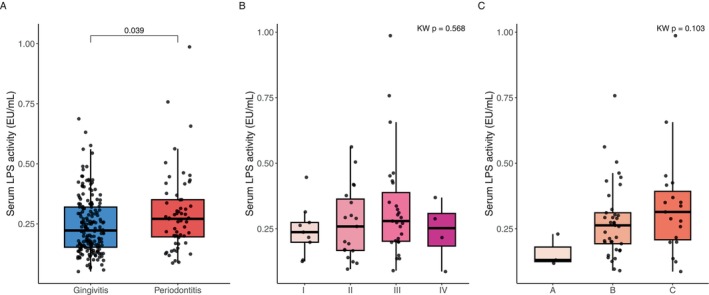
Serum LPS activity by periodontal status. (A–C) Comparisons by diagnosis, stage and grade, respectively. Values above plots indicate *p*‐values from non‐parametric tests (Wilcoxon rank‐sum for A; Kruskal–Wallis for B and C). Dots represent individual samples.

Serum LPS activity was significantly higher in the periodontitis group (median: 0.271 EU/mL; IQR: 0.196–0.351) than in the gingivitis group (0.222 EU/mL; IQR: 0.153–0.320) (*p* = 0.039) (Figure 1A). Serum LPS activity also showed a gradual increase from stage I to stage III and grade A to grade C of periodontitis, although these differences were not statistically significant (Figure 1B,C; Kruskal–Wallis *p* = 0.568 and *p* = 0.103, respectively).

Multivariate regression analysis (Table 2) revealed that higher serum LPS activity was associated with periodontitis diagnosis (*β* = 0.0482, 95% CI: 0.005–0.0914, *p* = 0.0291), even after adjustment for number and depth of periodontal pockets, FMBS, age, gender, diabetes, smoking status and BMI.

**TABLE 2:** Multivariate regression model identifying associations of periodontitis diagnosis with increased serum LPS activity, after adjustments for demographic and clinical variables.

Factors	Estimate (95% CI)	*p*
Periodontitis	0.0482 (0.005–0.0914)	**0.0291**
Number of periodontal pockets (4–5 mm)	0.0003 (−0.0028 to 0.0033)	0.8650
Number of periodontal pockets (≥ 6 mm)	−0.0004 (−0.0054 to 0.0046)	0.8662
FMBS	−0.0011 (−0.0026 to 4e‐04)	0.1360
Age	0.0001 (−0.0023 to 0.0025)	0.9946
Gender	0.0138 (−0.0244 to 0.052)	0.4786
Smoking	0.0221 (−0.0212 to 0.0654)	0.3157
Diabetes	−0.0481 (−0.1603 to 0.0641)	0.3990
BMI	−0.0001 (−0.0036 to 0.0033)	0.9454

*Note:* Size effects for categorical variables represent comparisons against the gingivitis group, female sex, non‐smoking status and absence of diabetes. Bold value indicates statistically significant.

### 3.3 Comparison of Subgingival LPS Activity, Bacterial DNA Load and Microbial Dysbiosis

**FIGURE 2:**
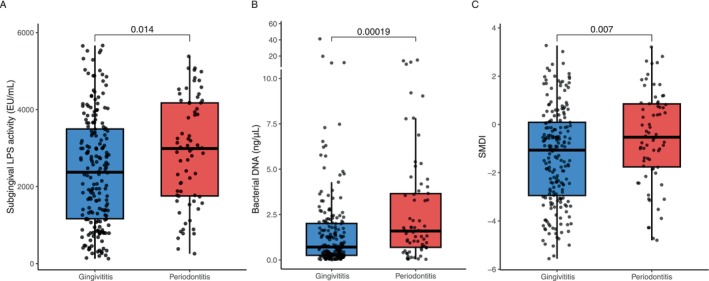
(A) Subgingival LPS activity, (B) bacterial DNA load and (C) subgingival microbial dysbiosis index (SMDI) compared between gingivitis and periodontitis patients.

The periodontitis group exhibited significantly higher subgingival LPS activity than the gingivitis group (median 2987.9 EU/mL, IQR: 1756.8–4172.6 vs. 2315.5 EU/mL, IQR: 1147.3–3442.8; *p* = 0.014) (Figure 2A). Within the periodontitis group, subgingival LPS activity differed significantly across stages and grades (*p* = 0.018 and *p* = 0.030, respectively), with higher values in more advanced disease, driven by differences between stage I and stages III and IV, and between grade A and grades B and C (Figure S2).

Subgingival bacterial DNA loads were also significantly higher in periodontitis (median 1.59 ng/μL, IQR: 0.70–3.65) compared with gingivitis participants (median 0.70 ng/μL, IQR: 0.25–2.06; *p* < 0.001; Figure 2B). In addition, SMDI indicated more pronounced dysbiosis in periodontitis, with significantly higher values compared to the gingivitis group (*p* = 0.007) (Figure 2C).

### 3.4 Subgingival Microbiome Characteristics

**FIGURE 3:**
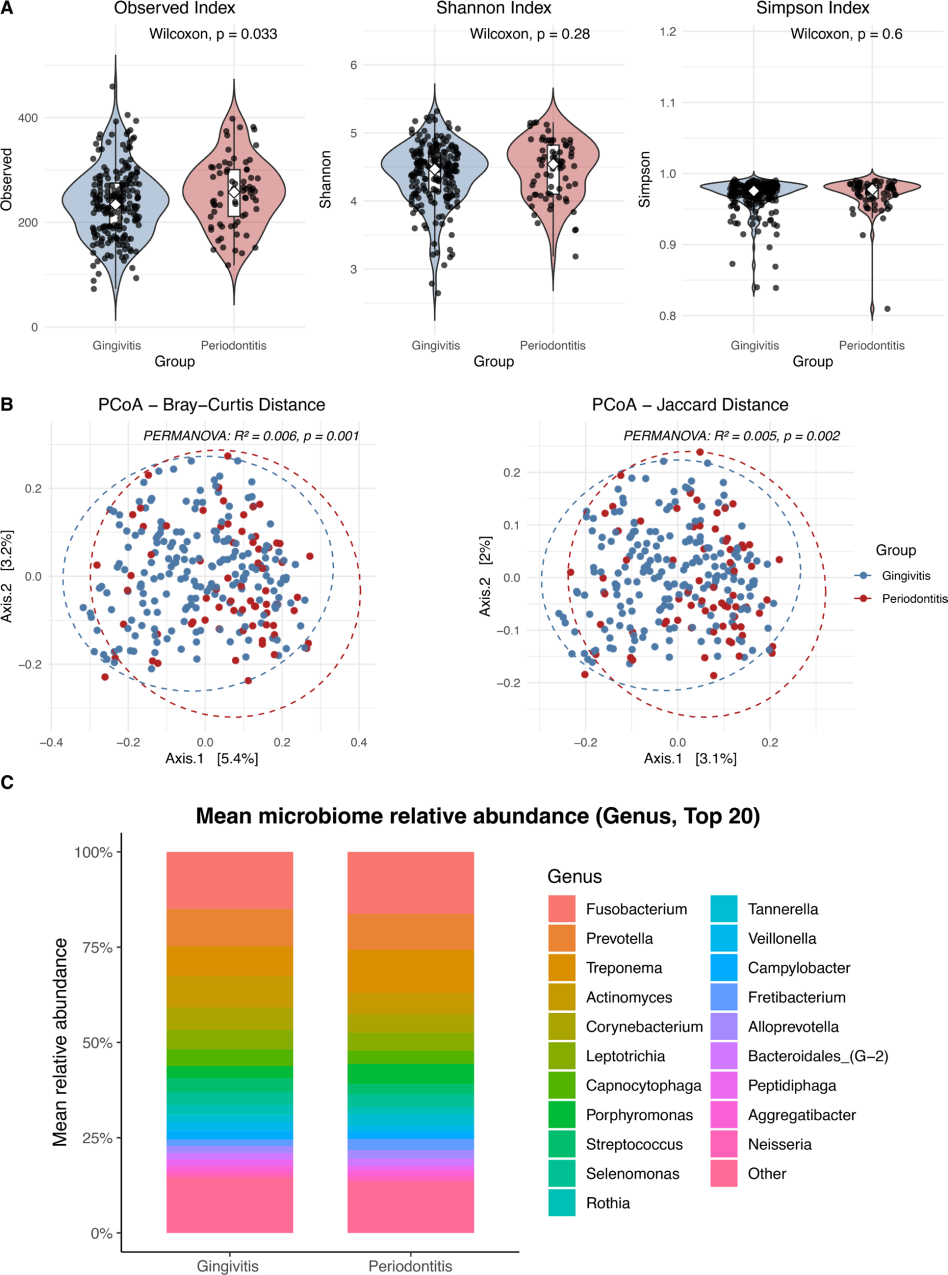
Alpha and beta diversity analyses comparing periodontitis and gingivitis groups: (A) Alpha diversity indices (observed, Shannon and Simpson) comparing gingivitis and periodontitis groups. (B) Beta diversity visualised by PCoA based on Bray–Curtis and Jaccard distances, with group differences tested using PERMANOVA. (C) Mean relative abundance profiles at the genus level in gingivitis and periodontitis groups.

Observed richness was higher in periodontitis (*p* = 0.033), whereas Shannon and Simpson indices showed no differences between periodontitis and gingivitis groups (*p* > 0.05) (Figure 3A). Principal coordinate analysis (PCoA) based on Bray–Curtis and Jaccard distances revealed distinct microbial clustering between the two groups, analysed by PERMANOVA test (Bray–Curtis: *R* ^2^ = 0.006, *p* = 0.001; Jaccard: *R* ^2^ = 0.005, *p* = 0.002), indicating differences in overall community composition (Figure 3B).

At the compositional level, genus‐level relative abundance profiles showed differences in dominant taxa between groups (Figure 3C), reflecting a shift towards disease‐associated genera, including *Porphyromonas*, *Treponema* and *Tannerella*, in periodontitis, whereas genera such as *Ottowia* and *Lactococcus* were more abundant in gingivitis. These patterns were supported by differential abundance analysis (Table S2).

### 3.5 Functional Pathways Differences Between the Groups

Functional prediction revealed that bacterial motility and colonisation, including flagellar assembly, bacterial chemotaxis, proteins involved in bacterial motility and glycan biosynthesis were significantly enriched in the periodontitis group. In contrast, pathways associated with host defence and cellular regulation, including RIG‐I‐like receptor signalling and apoptosis, were comparatively reduced in the periodontitis group. (Figure S3).

### 3.6 Correlations Between LPS Activities and Subgingival Microbiome

**FIGURE 4:**
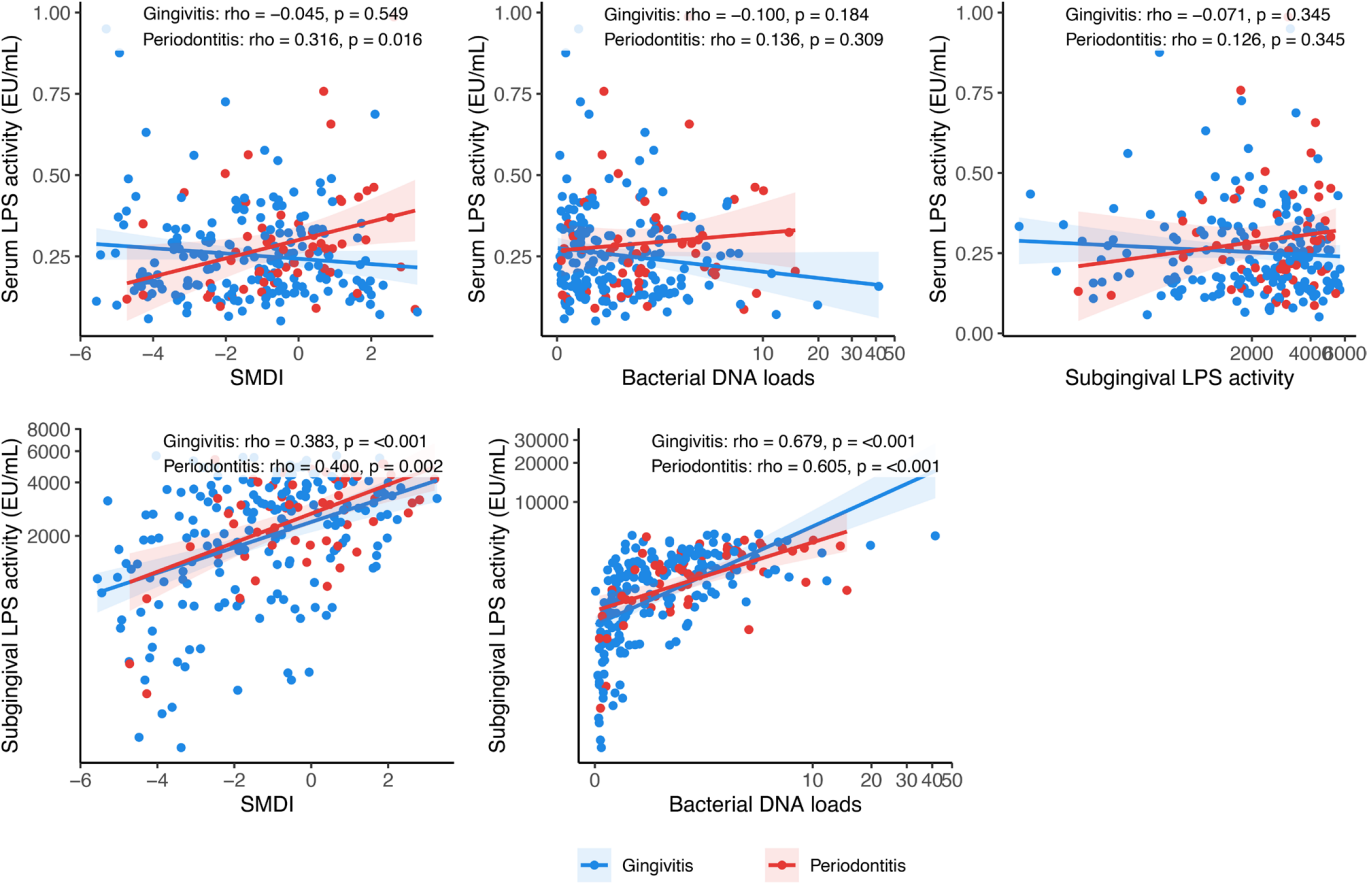
Group‐specific partial correlations between LPS activities and subgingival microbial parameters. Scatter plots show the associations between serum LPS activity or subgingival LPS activity and SMDI, bacterial DNA loads and subgingival LPS activity, stratified by gingivitis and periodontitis groups. Spearman's rank correlation coefficient (ρ) and corresponding *p*‐value were calculated on the original scale within each stratum and are annotated in each panel. Axes for skewed variables were displayed on a log‐transformed (log1p) scale. Solid lines indicate linear fits, and shaded areas represent 95% confidence intervals.

Serum LPS activity showed a statistically significant, moderate positive correlation with SMDI in the periodontitis group (*ρ* = 0.316, *p* = 0.016) (Figure 4). No significant associations were observed between serum LPS activity and subgingival bacterial DNA loads.

Subgingival LPS activity showed strong positive correlations with bacterial DNA loads in both groups (gingivitis: *ρ* = 0.679, *p* < 0.001; periodontitis: *ρ* = 0.605, *p* < 0.001) and moderate correlations with SMDI (gingivitis: *ρ* = 0.383, *p* < 0.001; periodontitis: *ρ* = 0.400, *p* = 0.002) (Figure 4). These associations remained significant even after adjustments for age, gender, smoking status, diabetes and BMI, using partial Spearman analyses (Table S3).

Correlations between serum and subgingival LPS activities were positive in the periodontitis group, but not statistically significant, while in the gingivitis group this association was negative (gingivitis: *ρ* = −0.071, *p* = 0.345; periodontitis: *ρ* = 0.126, *p* = 0.345; Figure 4 and Table S3). About 10% of serum LPS activity in the periodontitis group could be explained by subgingival LPS.

**FIGURE 5:**
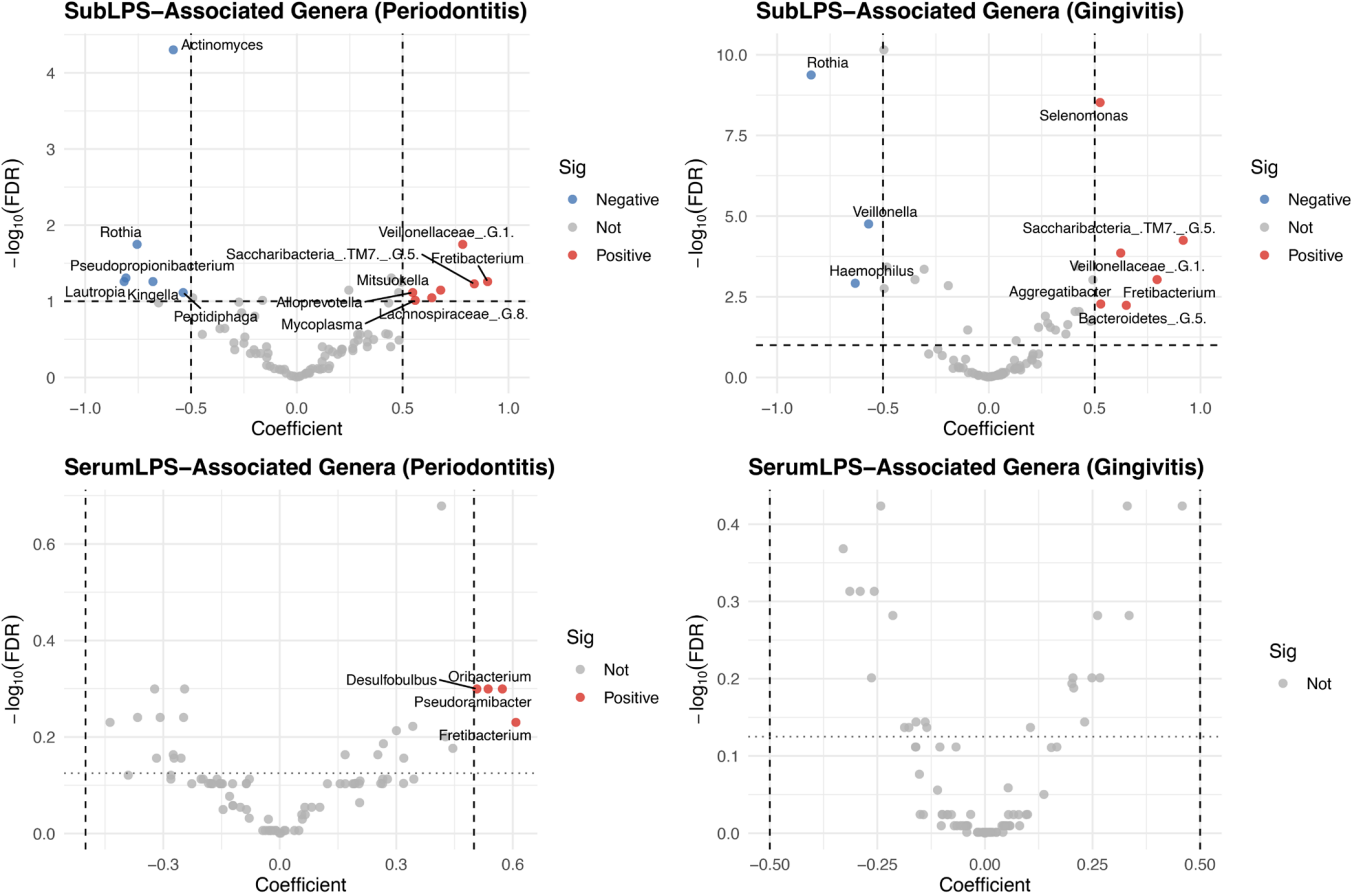
LPS‐associated genera identified by MaAsLin2. Volcano plots display genera associated with subgingival and serum LPS levels in periodontitis and gingivitis groups. MaAsLin2 linear models were adjusted for age, gender, smoking, diabetes and BMI. Genera with coefficients > |0.5| and FDR < 0.1 (for subgingival LPS) or any FDR (for serum LPS) are highlighted. Dashed lines indicate effect size thresholds (±0.5); dotted horizontal line in serum plots indicates FDR = 0.1.

MaAsLin2 identified multiple microbial genera significantly associated with LPS activity in both subgingival and serum compartments (Figure 5; Table S4).

In models adjusted for age, gender, smoking status, diabetes and BMI, subgingival LPS activity showed strong positive associations with *Fretibacterium*, *Saccharibacteria*_[TM7]_G‐5, *Veillonellaceae*_G‐1, *Mitsuokella, Lachnospiraceae*_G.8, *Mycoplasma* and *Alloprevotella* in the periodontitis group, while negative associations were observed with *Lautropia*, *Pseudopropionibacterium*, *Rothia*, *Kingella*, *Actinomyces* and *Peptidiphaga*. Similar trends were found in the gingivitis group, where *Saccharibacteria*_[TM7]_G‐5, *Fretibacterium*, *Bacteroidetes*_G‐5, *Veillonellaceae*_G‐1, *Aggregatibacter* and *Selenomonas* remained positively associated and *Rothia*, *Haemophilus* and *Veillonella* were negatively associated.

The associations between serum LPS activity and subgingival microbial genera were limited. In the periodontitis group, only the taxa *Pseudoramibacter*, *Desulfobulbus*, *Oribacterium* and *Fretibacterium* showed positive coefficients of > |0.5|, but none reached statistical significance (FDR > 0.7).

## 4 Discussion

This study comprehensively characterised serum and subgingival LPS activities in periodontitis patients, compared to gingivitis patients, and related it to clinical and subgingival microbiota attributes.

Gingivitis and periodontitis represent biologically distinct inflammatory states arising from dysbiotic biofilms. Gingivitis is a reversible, well‐controlled, precisely orchestrated and protective inflammatory response of the gums, whereas periodontitis is an irreversible, uncontrolled, destructive inflammatory disease and disruption of host homeostasis, affecting all supporting tissues (Darveau 2010).

Both subgingival and serum LPS activities demonstrated significantly higher levels in individuals with periodontitis compared to those with gingivitis. Periodontitis diagnosis was significantly associated with increased serum LPS activity, even after adjusting for other clinical and demographic parameters. It should also be noted that differences in age and sex distribution between groups may have influenced the observed associations, although these factors were adjusted for in the multivariable analyses. In addition, there was a gradual increase in serum LPS activity, correlated with higher stages (severity) and grades (rates of progression) of periodontitis.

Serum LPS was also associated with SMDI, in the periodontitis group only, but not with the overall bacterial burden. This association was absent in the gingivitis group, suggesting that the coupling between systemic LPS and dysbiosis emerges only once epithelial barrier integrity and protective inflammatory response are compromised. The correlation further implies that serum LPS reflects the influence of a specific subset of dysbiosis‐associated genera captured by SMDI, rather than total microbial burden. This observation is consistent with the concept that defined dysbiotic communities act as trans‐epithelial drivers of systemic immunogenic signals (Olsen and Singhrao 2018). These diagnosis‐specific associations highlight that systemic endotoxemia is not a passive by‐product of microbial presence but reflects host–microbial dysregulation with ecological and immunological specificity (Pietiäinen et al. 2018; Pussinen et al. 2022).

Subgingival LPS activity was most strongly correlated with subgingival bacterial DNA loads, while its correlations with SMDI were moderate, in both gingivitis and periodontitis groups. These associations underscore the potential of LPS as a functional readout of subgingival microbial community disturbances. This aligns with the observations of Chew et al. (2025), who found that SMDI was associated with NF‐κB activation, a pathway predominantly stimulated by LPS, suggesting a merger between microbial composition and inflammatory potential. The observed associations between LPS activity and bacterial DNA loads further reinforce the utility of subgingival LPS as an indicator of microbial burden and biofilm immunostimulatory potential (Dong et al. 2026).

Subgingival and serum LPS activities were not significantly correlated in either diagnostic group. Interestingly, the direction of association differed: being weakly negative in gingivitis and weakly positive in periodontitis, suggesting that 10% of serum LPS might be of periodontal origin. Although not statistically significant, this diagnosis‐specific divergence implies that systemic endotoxemia does not simply mirror local LPS levels, but rather represents a diagnosis‐dependent process influenced by epithelial barrier integrity and host regulatory mechanisms (Foroughi et al. 2025). These findings help explain why only a fraction of serum LPS could be assigned to subgingival sources, while other mucosal compartments, particularly the gastrointestinal tract, and systemic mechanisms likely contribute more to overall endotoxemia (Bajaj et al. 2018; Liljestrand et al. 2017). Serum LPS activities are shaped not only by local microbial burden but also by epithelial barrier function, host inflammatory status and systemic handling of endotoxin.

Functional prediction analysis supported a mechanistic link between subgingival dysbiosis and systemic endotoxemia. Periodontitis‐associated communities were enriched in motility and colonisation pathways, including flagellar assembly and chemotaxis, consistent with enhanced tissue penetration and dissemination of bacterial products such as LPS (Enersen et al. 2013). Concurrent depletion of pathways related to host defence and epithelial barrier regulation suggests impaired local immune surveillance that may facilitate endotoxin translocation. Together, these findings indicate that periodontitis‐associated dysbiosis is functionally adapted to promote LPS dissemination and systemic immunogenic signalling (Kumar et al. 2024; Santos et al. 2025).

*Fretibacterium* was significantly enriched in periodontitis and correlated with both subgingival and serum LPS activities. As a recognised core genus in periodontitis, it has been linked with inflammatory biofilms (Oh et al. 2024) and induces IL‐1β and TNF‐α in macrophages (Lafleur et al. 2023), likely via lipid A‐mediated mechanisms. Co‐occurrence with other LPS‐producing taxa may amplify TLR4‐mediated endotoxin signalling, particularly in deep pockets with impaired barriers (Lin et al. 2021). Similar LPS‐related associations were observed in salivary datasets, where *Prevotella, Leptotrichia* and *Porphyromonas* contributed most to endotoxin activity (Manzoor et al. 2026).

In addition to *Fretibacterium*, a subset of dysbiosis‐associated taxa, including *Saccharibacteria_TM7.G5*, *Veillonellaceae_G1* and *Mitsuokella*, have been implicated in epithelial disruption and dysregulated immune responses (Chouhan et al. 2025; Scheiman et al. 2019). These taxa may further influence community‐level metabolic activity and virulence, potentially enhancing the release and immunogenicity of LPS and facilitating its translocation across compromised epithelial barriers (Sharma et al. 2022; Zenobia and Darveau 2022).

In contrast, genera negatively associated with LPS activity, including *Actinomyces*, *Rothia*, *Veillonella* and *Lautropia*, were enriched in individuals with gingivitis and may play a protective role in maintaining immune homeostasis (Mazurel et al. 2023; Vielkind et al. 2015). Their inverse association with LPS likely reflects both ecological displacement and loss of protective function during dysbiosis.

These findings support the notion that specific genera within the subgingival microbiome influence both local LPS accumulation and systemic exposure. The overlap between LPS‐associated taxa and those discriminative of periodontal status reinforces their pathobiological relevance. These microbes may drive systemic endotoxemia via multiple routes: enhanced LPS biosynthesis, increased epithelial permeability, or via extracellular vesicles (Hajishengallis et al. 2023). Their identification not only deepens our mechanistic understanding of the oral‐systemic interface but also nominates potential microbial targets for diagnostic and therapeutic innovation in periodontitis‐associated systemic diseases.

This study integrated both subgingival and serum LPS activity data, enabling a multidimensional assessment of the local–systemic immune interface. To our knowledge, no studies have simultaneously quantified these two compartments while investigating their microbial correlations. The inclusion of LPS‐associated taxa provides novel insight into the ecological drivers of endotoxemia in periodontitis.

These findings offer new insights into the interplay between periodontal microbiota and systemic inflammatory burden, suggesting that local microbial signatures may hold broader clinical relevance beyond oral health (Pussinen et al. 2022). Nevertheless, the cross‐sectional design limits causal inference regarding the directionality between LPS activity and systemic inflammation. Subgingival sampling was performed following periodontal probing and may have influenced the local microbial composition due to mechanical disruption of the biofilm. In addition, the full continuum from periodontal health to disease was not represented, as analyses were restricted to the gingivitis and periodontitis groups. Future mechanistic studies should explore how specific microbes facilitate endotoxin translocation across the gingival epithelium. Cellular‐level investigations into epithelial barrier integrity, immune receptor activation and vesicle‐mediated transport may elucidate pathways through which subgingival dysbiosis contributes to systemic endotoxemia. Such insights could support the integration of microbial and immunological parameters into predictive tools for personalised periodontal care.

## 5 Conclusion

This study provides an integrated assessment of the link between serum LPS activity, periodontitis and subgingival dysbiosis. By discerning the association between increased serum LPS activity, periodontitis diagnosis, subgingival dysbiosis and specific microbial taxa and functional pathways, our findings highlight potential mechanisms by which periodontitis may influence systemic inflammatory burden. The identification of LPS‐associated genera that overlap with disease‐enriched taxa strengthens the hypothesis that specific bacteria contribute to oral–systemic immune‐inflammatory crosstalk. Furthermore, subgingival LPS activity emerged as a functional, microbiota‐derived marker of dysbiosis, reflecting both compositional imbalance and heightened immunostimulatory potential within the subgingival biofilm.

## Author Contributions

Anbo Dong contributed to the conception, design, data acquisition and interpretation, performed all statistical analyses and drafted and critically revised the manuscript. Muhammed Manzoor and Jaakko Leskelä contributed to the conception, data acquisition and interpretation, and critically revised the manuscript. Jukka Putaala, Eija Könönen and Yi Jin contributed to data acquisition and critically revised the manuscript. Susanna Paju and Pirkko Pussinen contributed to the conception, design, data acquisition and interpretation, and critically revised the manuscript. Svetislav Zaric contributed to the conception, design, data acquisition and interpretation and analysis, and drafted and critically revised the manuscript. All authors gave their final approval and agree to be accountable for all aspects of the work.

## Funding

This study was supported by the Academy of Medical Sciences (SGL023/1035 to S.Z.), Medical Research Council (MRC) Impact Acceleration Accounts (IAAs), King's College London (MR/X502923/1), Engineering and Physical Sciences Research Council (EPSRC) (EP/X525571/1) and the King's–China Scholarship Council PhD Scholarship Programme (202108410182 to A.D.). Additional support was provided by the Centre for Host‐Microbiome Interactions, Faculty of Dentistry, Oral & Craniofacial Sciences, King's College London. The SECRETO Oral study was funded by the Research Council of Finland (316777 and 355532 to S.P., 340750 to P.J.P.), the Finnish Dental Society Apollonia (to P.J.P.) and the Sigrid Jusélius Foundation (to P.J.P.). The parent SECRETO study was supported by the Research Council of Finland (286246, 318075, 322656 to J.P.), the Helsinki and Uusimaa Hospital District (TYH2014407, TYH2018318 to J.P.), the Sigrid Jusélius Foundation (to J.P.) and the Finnish Medical Foundation (to J.P.).

## Conflicts of Interest

The authors declare no conflicts of interest.

## Supporting information


**Data S1:** Supplementary methods: Detailed descriptions of DNA extraction, 16S rRNA sequencing, bioinformatic processing and functional inference analyses.
**Figure S1:** Flowchart of sample selection and exclusions in the SECRETO Oral cohort.
**Figure S2:** Distribution of subgingival LPS activities across periodontitis stages and grades.
**Figure S3:** Differential microbial functional pathways between periodontitis and gingivitis groups predicted by PICRUSt2.
**Table S1:** Genera included in the calculation of the subgingival microbial dysbiosis index (SMDI).
**Table S2:** Differentially abundant genera between gingivitis and periodontitis groups.
**Table S3:** Correlations between serum and subgingival LPS activities and microbial parameters using unadjusted and adjusted Spearman analyses.
**Table S4:** Genera associated with subgingival and serum LPS activities identified by MaAsLin2 analyses.

## Data Availability

The data that support the findings of this study are openly available in the European Nucleotide Archive (ENA) at https://www.ebi.ac.uk/ena/browser/view/PRJEB82979.
