# Supplementary material for: Periodontitis and Endotoxemia: Clinical and Microbiological Insights
Source: J Clin Periodontol. 2026 Jun 4;53(8):1344–54. doi: 10.1111/jcpe.70148 (PMC13371422; doi:10.1111/jcpe.70148)
Supplement: Supplementary file 1 — Data S1: Supplementary methods: Detailed descriptions of DNA extraction, 16S rRNA sequencing, bioinformatic processing and functional inference analyses. Figure S1: Flowchart of sample selection and exclusions in the SECRETO Oral cohort. Figure S2: Distribution of subgingival LPS activities across periodontitis stages and grades. Figure S3: Differential microbial functional pathways between periodontitis and gingivitis groups predicted by PICRUSt2. Table S1: Genera included in the calculation of the subgingival microbial dysbiosis index (SMDI). Table S2: Differentially abundant genera between gingivitis and periodontitis groups. Table S3: Correlations between serum and subgingival LPS activities and microbial parameters using unadjusted and adjusted Spearman analyses. Table S4: Genera associated with subgingival and serum LPS activities identified by MaAsLin2 analyses. [file JCPE-53-1344-s001.docx]

**Periodontitis and Endotoxemia: Clinical and Microbiological Insights**

Anbo Dong, Muhammed Manzoor, Jaakko Leskelä, Jukka Putaala, Eija Kononen, Yi Jin, Pirkko Pussinen, Susanna Paju, Svetislav Zaric

***Supplementary* *Methods***

- 1. **DNA Extraction and Sequencing**
     DNA was extracted using the Chemagic Saliva600 protocol (PerkinElmer) with automated purification. Total bacterial DNA was quantified by qPCR using the Femto™ Bacterial DNA Quantification Kit (Zymo Research) to estimate microbial load. The V1–V2 region of the 16S rRNA gene was amplified with primers 27F-YM (AGT CAG TCT GTC AGA GTT TGA TYM TGG CTC AG) and 338R-R (TAT GGT AAT TCA TGC TGC CTC CCG TAG RAGT) (Frank et al., 2008) using Phusion Green Hot Start II High-Fidelity PCR Master Mix (Thermo Fisher Scientific) on a Bio-Rad C1000 Touch thermal cycler. Amplicons were purified with the QIAquick PCR Purification Kit (QIAGEN), quantified by PicoGreen dsDNA assay (Thermo Fisher Scientific), and sequenced using 250 bp paired-end chemistry on the Illumina MiSeq platform at the Queen Mary University of London Genome Centre.
  2. **Bioinformatic Processing**
     Raw reads were processed in R using DADA2 (v1.14.1). Forward and reverse reads were quality-filtered and trimmed to 200 bp and 160 bp, respectively. Error models were inferred, amplicon sequence variants (ASVs) were generated, paired-end reads were merged, and chimeras removed. Taxonomy was assigned using the Human Oral Microbiome Database (HOMD v15.1).
  3. **Functional Inference**
     Predicted functional profiles were generated with PICRUSt2 (v2.5.2) (Douglas et al., 2020). Normalised ASV tables were used to infer KEGG orthologs (KOs), which were collapsed into higher-level KEGG pathways. Pathway abundances were centre log-ratio (CLR) transformed before testing. Group differences were evaluated using Wilcoxon rank-sum tests with Benjamini–Hochberg false discovery rate (FDR) correction; pathways with FDR ≤ 0.25 and raw p < 0.05 were considered significant.

***Supplementary Figure S1. Flowchart of sample selection and exclusions in the SECRETO Oral cohort.*** Of the 329 participants originally recruited in the SECRETO Oral study, subgingival plaque samples were successfully collected from 324 participants. Among these, 290 yielded valid DNA extraction and 16S rRNA sequencing data. After excluding 19 participants due to recent antibiotic use and one periodontally healthy participant, 270 participants were included in the final microbiome and LPS analyses.

***Supplementary Table S1. List of discriminating genera used for the calculation of the subgingival microbiome dysbiosis index (SMDI).***

| SMDI | |
| --- | --- |
| Type | **Name** |
| Dysbiosis | *Fretibacterium* |
|  | *Treponema* |
|  | *Mogibacterium* |
|  | *Peptostreptococcaceae [XI][G-6]* |
|  | *Desulfobulbus* |
|  | *Peptostreptococcaceae [XI][G-5]* |
|  | *Tannerella* |
|  | *Filifactor* |
|  | *Peptostreptococcaceae [XI][G-4]* |
|  | *Lachnospiraceae [G-8]* |
|  | *Bacteroidaceae [G-1]* |
|  | *Bacteroidetes [G-3]* |
| Normobiosis | *Actinomyces* |
|  | *Streptococcus* |
|  | *Comamonas* |
|  | *Rothia* |
|  | *Gemella* |
|  | *Granulicatella* |
|  | *Corynebacterium* |

*Note: The subgingival microbiome dysbiosis index (SMDI) was calculated as the difference in the mean CLR-transformed abundance between predefined dysbiotic and normobiotic genera. Higher values of both indices reflect a greater degree of microbial imbalance within the subgingival biofilm. The genera used for these calculations were selected based on previously validated discriminatory profiles.*

***Supplementary Figure S2. Distribution of subgingival LPS activities across periodontitis stages and grades.*** ***Boxplots showing the distribution of subgingival lipopolysaccharide (LPS) activities among patients with periodontitis, stratified by (A) stage and (B) grade***

***Supplementary Table S2. Differentially abundant genera between gingivitis and periodontitis.*** *Genus-level differential abundance analysis was performed with adjustment for age, sex, smoking, diabetes, and BMI. LogFC values represent enrichment in periodontitis (positive) or gingivitis (negative). P values were adjusted using the Benjamini–Hochberg method (FDR). Only selected taxa with FDR < 0.05 are shown*

| Taxon | logFC | FDR | Direction |
| --- | --- | --- | --- |
| *Ottowia* | -0.959 | 0.000 | Enriched in Gingivitis |
| *Desulfovibrio* | -0.583 | 0.022 | Enriched in Gingivitis |
| *Acidipropionibacterium* | -0.545 | 0.024 | Enriched in Gingivitis |
| *Lactococcus* | -0.447 | 0.020 | Enriched in Gingivitis |
| *Gracilibacteria_(GN02)_(G-1)* | 0.427 | 0.028 | Enriched in Periodontitis |
| *Peptoniphilus* | 0.429 | 0.029 | Enriched in Periodontitis |
| *Lactobacillus* | 0.477 | 0.029 | Enriched in Periodontitis |
| *Clostridiales_(F-1)(G-1)* | 0.514 | 0.017 | Enriched in Periodontitis |
| *Peptostreptococcaceae_(XI)(G-4)* | 0.516 | 0.011 | Enriched in Periodontitis |
| *Filifactor* | 0.536 | 0.007 | Enriched in Periodontitis |
| *Peptostreptococcaceae_(XI)(G-5)* | 0.557 | 0.012 | Enriched in Periodontitis |
| *Mycoplasma* | 0.578 | 0.028 | Enriched in Periodontitis |
| *Bacteroidales_(G-2)* | 0.614 | 0.029 | Enriched in Periodontitis |
| *Lachnospiraceae_(G-8)* | 0.625 | 0.005 | Enriched in Periodontitis |
| *Ruminococcaceae_(G-2)* | 0.635 | 0.001 | Enriched in Periodontitis |
| *Bacteroidaceae_(G-1)* | 0.713 | 0.001 | Enriched in Periodontitis |
| *Porphyromonas* | 0.741 | 0.018 | Enriched in Periodontitis |
| *Treponema* | 0.752 | 0.020 | Enriched in Periodontitis |
| *Tannerella* | 0.789 | 0.004 | Enriched in Periodontitis |
| *Fretibacterium* | 0.825 | 0.011 | Enriched in Periodontitis |
| *Leptothrix* | 1.073 | 0.000 | Enriched in Periodontitis |

***Supplementary Figure* S3. Differential microbial functional pathways between periodontitis and gingivitis.** Note: KEGG pathways predicted by PICRUSt2 with significant differences between periodontitis and gingivitis groups (FDR ≤ 0.25, p < 0.05).

***Supplementary Table S3. Correlations of serum and subgingival LPS activities with subgingival microbial parameters using unadjusted and covariate-adjusted Spearman analyses.***

|  | | Serum LPS | | | | Subgingival LPS | | | |
| --- | --- | --- | --- | --- | --- | --- | --- | --- | --- |
|  |  | Unadjusted Spearman | | Partial Spearman | | Unadjusted Spearman | | Partial Spearman | |
| Variable | Group | Rho | p | Rho | p | Rho | p | Rho | p |
| Bacterial DNA Loads | Periodontitis | 0.136 | 0.309 | 0.065 | 0.626 | 0.605 | **<0.001** | 0.561 | **<0.001** |
|  | Gingivitis | -0.100 | 0.184 | -0.104 | 0.164 | 0.679 | **<0.001** | 0.666 | **<0.001** |
| SMDI | Periodontitis | **0.316** | **0.016** | **0.286** | **0.029** | 0.400 | **0.002** | 0.339 | **0.009** |
|  | Gingivitis | -0.045 | 0.557 | -0.047 | 0.531 | 0.383 | **<0.001** | 0.362 | **<0.001** |
| Subgingival LPS | Periodontitis | 0.126 | 0.345 | 0.069 | 0.607 |  | | | |
|  | Gingivitis | -0.071 | 0.345 | -0.088 | 0.239 |  |  |  |  |

***Supplementary* Table S4. Genera associated with subgingival and serum LPS levels in periodontitis and gingivitis patients.**

|  | Genus | Coef | FDR | Sig |
| --- | --- | --- | --- | --- |
| Subgingival LPS-Periodontitis patients | ***Fretibacterium*** | 0.9015 | 0.0551 | Positive |
|  | ***Saccharibacteria_.TM7._.G.5.*** | 0.8385 | 0.0590 | Positive |
|  | ***Veillonellaceae_.G.1.*** | 0.7835 | 0.0179 | Positive |
|  | ***Mitsuokella*** | 0.6794 | 0.0714 | Positive |
|  | ***Lachnospiraceae_.G.8.*** | 0.6377 | 0.0900 | Positive |
|  | ***Mycoplasma*** | 0.5592 | 0.0972 | Positive |
|  | ***Alloprevotella*** | 0.5474 | 0.0768 | Positive |
|  | ***Peptidiphaga*** | -0.5370 | 0.0768 | Negative |
|  | ***Actinomyces*** | -0.5838 | <0.0001 | Negative |
|  | ***Kingella*** | -0.6801 | 0.0551 | Negative |
|  | ***Rothia*** | -0.7549 | 0.0179 | Negative |
|  | ***Pseudopropionibacterium*** | -0.8076 | 0.0494 | Negative |
|  | ***Lautropia*** | -0.8160 | 0.0551 | Negative |
| Subgingival LPS-Gingivitis | ***Saccharibacteria_.TM7._.G.5.*** | 0.9163 | 0.0001 | Positive |
|  | ***Fretibacterium*** | 0.7910 | 0.0009 | Positive |
|  | ***Bacteroidetes_.G.5.*** | 0.6518 | 0.0055 | Positive |
|  | ***Veillonellaceae_.G.1.*** | 0.6202 | 0.0001 | Positive |
|  | ***Aggregatibacter*** | 0.5340 | 0.0052 | Positive |
|  | ***Selenomonas*** | 0.5253 | <0.0001 | Positive |
|  | ***Veillonella*** | -0.5663 | <0.0001 | Negative |
|  | ***Haemophilus*** | -0.6310 | 0.0011 | Negative |
|  | ***Rothia*** | -0.8399 | <0.0001 | Negative |
| Serum LPS-tis | ***Pseudoramibacter*** | 0.6198 | 0.7510 | Positive |
|  | ***Desulfobulbus*** | 0.5478 | 0.7510 | Positive |
|  | ***Oribacterium*** | 0.5285 | 0.7510 | Positive |
|  | ***Fretibacterium*** | 0.5283 | 0.8154 | Positive |

The table summarises the genera significantly associated with LPS levels identified via MaAsLin2 multivariate linear modelling. Subgingival LPS (gingivitis and periodontitis patients were analysed separately) and for serum LPS correlations were analysed only in periodontitis patients. Genera were considered associated if they showed a coefficient (Coef) > |0.5| and a false discovery rate (FDR) < 0.1 (subgingival). Genus-level associations are listed alongside their effect size (Coef), FDR-adjusted p-values, and direction of association (Positive/Negative). Abbreviations: Coef, regression coefficient; FDR, false discovery rate; Sig, significance direction;

**Reference:**

Douglas, G. M., Maffei, V. J., Zaneveld, J. R., Yurgel, S. N., Brown, J. R., Taylor, C. M., Huttenhower, C., & Langille, M. G. I. (2020). PICRUSt2 for prediction of metagenome functions. *Nature Biotechnology*, *38*(6), 685–688. <https://doi.org/10.1038/s41587-020-0548-6>
